# Supplementary material for: Pathogenic Leptospira Infections in Hubei Province, Central China
Source: Microorganisms. 2022 Dec 30;11(1):99. doi: 10.3390/microorganisms11010099 (PMC9865294; doi:10.3390/microorganisms11010099)
Supplement: Supplementary file 1 [file microorganisms-11-00099-s001.zip › microorganisms-2114793-supplementary.pdf]

Supplementary Materials

Table S1. Primers used in this study.

Gene	Primers	Sequences(5’-3’)	Amplicon	Reference
rrs	*rrs*-1F	GGCGGCGCGTCTTAAACATG	1100	[20]
	rrs-2F	CAAGTCAAGCGGAGTAGCAA		[20]
	rrs-1R	GTACAAGGTCCGGGAACGTA		[20]
	rrs-2R	GCGAGTTGGCTACCCTTTGT		[20]
LipL32	*LipL32*-1F	ATCTCCGYTGCACTCTTTGCA	730	This study
	LipL32	TCTTCRGCDGCKATAGCTTG		This study
	LipL32-2R	CARTTCTTCAGGATTTGAGTGG		This study
secY	*secY*-F	GAAGGWCTTCTCGGAATGGTGG	1200	[20]
	secY-1R	CCKTCCCTTAATTTTAGACTTCTTC		[20]
	secY-2R	TTCATRAAGCCTTCRTAATTTCTCA		[20]
